# PREVALENCE OF HUMAN PARVOVIRUS B19 IgG AND IgM ANTIBODIES AMONG PREGNANT WOMEN ATTENDING ANTENATAL CLINIC AT FEDERAL TEACHING HOSPITAL IDO-EKITI, NIGERIA

**DOI:** 10.21010/ajid.v15i2.3

**Published:** 2021-03-18

**Authors:** Richard Yomi Akele, Jennifer Tamuno Abelekum, Bernard Oluwapelumi Oluboyo, Janet Funmilayo Akinseye, Seyi Samson Enitan, Olusola Ayodeji Olayanju, Emmanuel Jide Akele

**Affiliations:** 1 Department of Medical Laboratory Science, Afe Babalola University, Ado-Ekiti, Nigeria; 2 Department of Medical Laboratory Science, Babcock University, Ilishan-Remo, Nigeria

**Keywords:** Antibodies, Human Parvovirus B19, IgM, IgG, Pregnancy, Sero-prevalence, Nigeria

## Abstract

**Background:**

Human Parvovirus *B19* (*B19V*) is a DNA virus, transmitted through respiratory secretions, hand-to-mouth-contact, blood transfusion and trans-placental transfer. It causes high mortality and morbidity in pregnant women, thus contributing to poor maternal and child health. B19V has been neglected due to dearth of epidemiological data. The aim of this study was to determine the sero-prevalence of Human Parvovirus B19 antibodies among pregnant women attending antenatal clinic at Federal Teaching Hospital Ido-Ekiti, Nigeria.

**Materials and Methods:**

This cross-sectional study enrolled pregnant women attending Federal Teaching Hospital Ido-Ekiti from January to May 2019 to obtain sero-epidemiological data. One hundred and twenty-two (122) consenting pregnant women were enrolled following institutional ethical approval. They were administered structured questionnaire and venous blood was collected in plain tubes for serum extraction. Sera samples were analyzed for IgG and IgM antibodies using the enzyme linked immunosorbent assay method. Percentages, median, chi-square and multivariate analysis were carried out using SPSS version 17.

**Results:**

The prevalence of IgG was 44.3% (54/122), IgM 41.8% (51/122), and IgG/IgM 28.7% (35/122) leaving 55.7% (68/122) of the population uninfected. The median age of participants was 22 (Interquartile range 18-25) years among which 36-45years had the highest prevalence which was not statistically significant (p=0.09 *X*^2^ =4.75). There was association between miscarriage, still birth, history of blood transfusion and prevalence of *Human Parvovirus B19* (p<0.05).

**Conclusion:**

There is a high Prevalence of B19V among pregnant women attending antenatal clinic in this study. This underscores the need for testing and immunization of pregnant women against B19V.

## Introduction

Human Parvovirus B19 (B19V) is a 5.5kb single stranded, non-enveloped DNA virus, transmitted through respiratory secretions, hand to mouth contact, blood transfusion and trans-placental transfer (Gratacós *et al.*, 1995). *B19V* has been linked as a cause of hydrops fetalis, intrauterine fetal death, aplastic crisis, spontaneous abortion, acute symmetric polyarthropathy and erythema infectiosum (Fifth disease) (Rodis *et al.*, 1988; Gratacós *et al.*,1995; Simchen *et al.*, 2002). Its discovery was first documented in 1975 (Cossart *et al.*, 1975).

Human Parvovirus B19 infects erythrocyte progenitor cell stimulating apoptosis of the infected cells. Infections with this virus are usually asymptomatic in most patients but acute infection in patients with a high erythrocyte turnover may lead to a life-threatening aplastic crisis. For instance, acute congenital *B19V* infection in pregnancy results in non-immune hydrops fetalis (NIHF) and fetal death (Juhl and Henning, 2018). Fetuses at first and second trimester are most susceptible because erythroid precursor development is predominant at these stages (Heegaard and Brown, 2002). Of the three genotypes described worldwide, type 3 is reported to be the most commonly occurring in the North and West Africa (Blumel *et al.*, 2012). *B19V* has specific tropism for blood group Antigen P (globosid) which is expressed on the surface of erythrocyte precursor cells, megakaryocytes, endothelia cells, placenta cells and cells at fetal myocardium (Juhl and Henning, 2018). 

Though the prevalence of *B19V* among pregnant women in developing countries is not well documented, few available documents show a sero-prevalence of 3.3% for *B19V* IgM in South Africa (Mirambo *et al.*, 2017), 32.8% in Tanzania (Mirambo *et al.*, 2017) and 13.2% in Central Nigeria (Emiasegen *et al.*, 2011). A prevalence of 24.9% for *B19V* IgG has been documented in South Africa, 55.0% in Tanzania (Mirambo *et al.*, 2017), 27.5% in Central Nigeria (Emiasegen *et al.*, 2011) and 20% in South-West Nigeria (Abiodun *et al.*, 2013).

Nigeria has a high maternal and child mortality rate which has become the main focus of the government in health care delivery in recent years. A number of causes for these mortalities have been listed; unfortunately, *B19V* despite its immense impact on child and maternal mortality was not enlisted (Abiodun *et al.*, 2013). This is possibly due to paucity of epidemiological data on impact of *B19V* on pregnant women in Nigeria. The aim of this study was to determine the prevalence and impact of Parvovirus B19V in pregnant women.

## Materials and Methods

### Study area

This cross-sectional study was conducted between January and May 2019 at the Federal Teaching Hospital Ido-Ekiti (FETHI), Ekiti State, among pregnant women attending antenatal clinic. Federal Teaching Hospital Ido-Ekiti is a referral hospital for Ado-Ekiti and some towns in neighboring states such as Kogi, Kwara and Osun states. It is located in Ido-Osi Local Government Area of Ekiti State Nigeria, latitude 7.843093, longitude 5.182314 ..\Documents\Find coordinates.docx. Laboratory analysis was carried out in the Medical Laboratories of the Department of Medical Laboratory Science, Afe Babalola University, Ado-Ekiti (ABUAD), Ekiti State. Ado-Ekiti is a city in Southwestern Nigeria and lies on latitude 7^o^ 35 and 7^o^38 north of the equator and longitude 5^o^10 and 5^o^15 east of the Greenwich Meridian (Jameson *et al.*, 1995). Afe Babalola University is a private institution with its campus located at Km. 8.5, Afe Babalola way, opposite Federal Polytechnic, Ado-Ekiti.

### Study Population/Design

The study population included one hundred and twenty-two pregnant women attending antenatal clinic at the Federal Teaching Hospital Ido-Ekiti. Subjects who consented to take part in the study were randomly selected and administered a structured questionnaire to collect social demographic and some health history data.

### Ethical Consideration

Ethical approval was obtained in accordance with the Helsinki declaration from the Ethics and Research Committee, Federal Teaching Hospital, Ido-Ekiti, Ekiti State (Protocol No: ERC/2019/03/13/199B). The study participants were informed about the purpose of the study and written consent was obtained from each participant before sample collection.

### Inclusion/Exclusion Criteria

- Pregnant women at exactly their 36^th^ week were excluded.
- Those with gestational diabetes and high blood pressure were excluded.

### Sample size

The minimum sample size for this study was calculated using Yamane Taro’s formula (1967) for finite population (Singh and Masuku, 2014).

Where,

n= minimum sample size

N= average number of pregnant women attending FTH IDO-EKITI in six months

e= level of precision

N= 198

e= 0.05


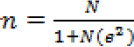



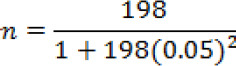



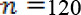


### Sample collection

One hundred and twenty-two (122) 5ml venous blood samples were obtained from consenting participants after the administration of a structured questionnaire into plain tubes. Samples were centrifuged at 1000 rpm (revolution per minute) for 10 minutes. The Sera were harvested using Eppendorf tubes and stored at -20°C (Cheesbrough, 2006).

### Detection Parvovirus B19 IgG and IgM antibodies.

The sera samples were used to detect parvovirus B19V IgG and IgM antibodies using ELISA kit (Melsin Medical CO Ltd, China): according to manufacturer’s instructions.

### Test procedure for IgG/IgM 

Briefly, the test procedures were as follows: The reagents provided were allowed to attain room temperature for 15 minutes before use. The 20X wash buffer was diluted with distilled water using a ratio of 1:20 before use. The micro-titer plate template was set up with 1 well as blank, 2 wells as negative control and 2 wells as the positive control. 10µl of sera sample and 40µl sample diluent were dispensed into the respective wells except for the blank well, negative control well and positive control well. 50 µl of the negative and positive controls were dispensed into their wells respectively. The content was mixed by vibrating the plate gently. The microplate was covered with a sealing paper and incubated in a microplate incubator (MARVOTECH plate incubator, China) at 37^o^C for 60 minutes (Akele *et al.*, 2020a). After incubation, the microplate was washed five times using wash buffer. 100 µl of Horseradish peroxidase enzyme (HRP) conjugate was added to each well except the blank; the microplate was covered with a sealing paper and also incubated in a microplate incubator at 37^o^C for 15 minutes. After incubation, the microplate was washed five times with the diluted wash buffer in an automatic plate washer (MARVOTECH plate washer, China) (Akele *et al.*, 2020b). 50 µl of substrate solution A and B were added to each well respectively and were mixed; the plate was covered and incubated at 37^o^C for 15 minutes. 50 µl of stop solution was added to each well and mixed. The absorbance was read in an ELISA reader machine (MARVOTECH ELISA reader, China) at a wavelength of 450 nm (Oluboyo *et al.*, 2019).

### Interpretation of results

If mean negative control O.D < 0.1 and the mean positive O.D > 0.8, the test is valid.

Cut off = the mean O.D value of the negative control × 2.1

Positive results: Sample O.D > Cut-off O.D

Negative result: Sample O.D < Cut-off O.D

### Statistical analysis

Collected data were analyzed using statistical package for social science (SPSS) version 17. Categorical variables were summarized as proportions while continuous variables were summarized as median with interquartile range. A multivariate regression was also conducted to determine predictors of seropositivity of *B19V* in pregnant women.

## Results

Participants had a median age of 22(IQR 18-25) years. Out of the 122 subjects, 65 (53.3%) were age group 26-35 years. 11% and 55% of the subjects were in their first and second trimester respectively while the remaining 34% were in the third trimester. Twelve percent (12%) of them had a history of still birth or miscarriage. A total of 54/122 (44.3%) of subject were seropositive for *B19V* IgG antibodies while 51/122 (41.8%) were seropositive for *B19V* IgM antibodies leaving 68/122 (55.7%) as the susceptible population (Table 1). Thirty-five subjects (28.7%) were co-seropositive for both *B19V* IgG/IgM antibodies, while 13.1% (16/122) were seropositive for *B19V* IgM only (Table 2). Prevalence of IgG and IgM was higher among the 36-45years age group (9.0% and 7.4% respectively). However, there was no association between age and the distribution of *B19V* IgG, IgM and IgG/IgM antibodies (*p* = 0.09, 0.97 and 0.68 respectively).

**Table 1:** Prevalence of *Human Parvovirus B19* (*B19V*) IgG and IgM antibodies among pregnant women.

Age	B19V IgG				B19V IgM			
	
	Positive(%)	OR (95% CI)	P-Value	*χ^2^*	Positive(%)	OR (95% CI)	P-Value	*χ^2^*
**16-25**	11/122(9.0)	1			13/122(10.6)	1		
**26-35**	32/122(26.2)	1.9 (1.1-2.7)	0.09	4.75	29/122(23.7)	2.1 (0.9-3.1)	0.97	0.61
**36-45**	11/122(9.0)	1.1 (0.9-1.3)			9/122(7.4)	1.3 (0.7-1.5)		
**Total**	54/122(44.3)				51/122(41.8)			

OR=Odd ratio, CI=Confidence interval, X2 = Chi square, B19V=Human parvovirus B19

**Table 2:** *Human Parvovirus B19* (B19V) IgG/IgM Co-prevalence among pregnant women.

B19V IgG/IgM				

Age	Positive (%)	OR (95% CI)	P-Value	*χ^2^*
**16-25**	8/122(6.6)	1		
**26-35**	20/122(16.4)	1.5 (0.9-2.1)	0.68	6.13
**36-45**	7/122(5.7)	1.3 (0.9-1.7)		
**Total**	35/122(28.7)			

For the two vulnerable stages of pregnancy (first and second trimester), 4.1% (5/122) and 23% (28/122) respectively were seropositive for IgG while 3.3% (4/122) and 26.2% (32/122) respectively were seropositive for IgM antibodies leaving 44% of the population susceptible to *B19V*. There was no association between the stage of pregnancy and the distribution of *B19V* (*p* = 0.90 for IgG and 0.32 for IgM). There was an association between history of blood transfusion, complications and the seroprevalence of *B19V* as those who had history of blood transfusion (OR 2.1 95% CI 1.7 - 2.3) *p* = 0.05, miscarriages (OR 2.5 95% CI 1.7-3.3) and still births (OR 2.8 95% CI 1.7–3.1), *p* = 0.04 for IgG and (OR 2.1 95% CI 1.1–2.7) and (OR 1.7 95% CI 1.1–1.9) respectively, *p* = 0.05 for of IgM (Table 3). Parity, number of pregnancies, occupation and marital status hard no association with prevalence distribution of *B19V* (Table 3).

**Table 3:** Multivariate analysis of factors associated with *Human Parvovirus B19* (B19V) antibodies seropositivity.

B19V IgG					B19V IgM			

Factors	Positive (%)	OR (95% CI)	P- Value	*χ^2^*	Positive (%)	OR (95% CI)	P-Value	*χ^2^*
**Gestational Age**								
1^st^ Trimester	5/122(4.1)	1			4/122(3.3)	1		
2^nd^ Trimester	28/122(23.0)	1.1 (0.9-2.1)	0.90	0.64	32/122(26.2)	0.9(0.7-1.5)	0.32	2.27
3^rd^ Trimester	21/122(17.2)	1.7 (1.3-1.9)			15/122(12.3)	1.1(0.9-1.3)		
**Parity**								
None	23/122(18.9)	1			26/122(21.3)	1		
1-2	21/122(17.2)	1.2 (0.9-1.7)	0.77	1.13	18/122(14.8)	1.3(0.9-1.5)	0.99	0.14
3-4	9/122(7.3)	1.5 (1.3-1.7)			6/122(5.0)	1.1(0.9-1.3)		
5-6	1/122(0.8)	1.7 (1.5-2.1)			1/122(0.8)	0.9(0.7-1.3)		
**Number of pregnancy**								
1-2	25/122(20.5)	1			28/122(23.0)	1		
3-4	24/122(19.7)	1.5 (1.1-1.7)	0.06	0.09	20/122(16.4)	0.9(0.7-1.1)	0.63	1.72
5-6	5/122(4.1)	2.0 (1.7-2.3)			4/122(3.3)	0.8(0.7-1.1)		
**History of Blood Transfusion**								
No	53/122(43.4)	1			50/122(41.0)	1	0.05	0.76
Yes	1/122(0.8)	2.1(1.7-2.3)	0.21	0.04	1/122(0.8)	2.5 (1.8-2.9)		
**Occupation**								
Student	3/122(2.4)	1			6/122(5.0)	1		
Self employed	32/122(26.2)	2.1 (1.7-2.3)	0.13	5.67	30/122(24.6)	0.9(0.6-1.1)	0.14	0.99
House wife	11/122(9.0)	1.5 (1.3-1.7)			7/122(5.7)	0.9(0.7-1.1)		
Civil servant	8/122(6.6)	1.7 (1.5-2.1)			8/122(6.6)	0.7(0.6-1.3)		
**Marital Status**								
Married	50/122(41)	1	0.42	1.73	45/122(36.9)	1	0.83	0.37
Single	4/122(3.3)	0.6 (0.5-1.0)			6/122(5.0)	1.3(1.1-1.5)		
**Complication History**								
None	38/122(31.1)	1			41/122(33.6)	1		
Miscarriage	13/122(10.7)	2.5 (1.7-3.3)	0.04	0.85	11/122(9.0)	2.1(1.1-2.7)	0.05	2.27
Still birth	2/122(1.6)	2.8 (1.7-3.1)			1/122(0.8)	1.7(1.1-1.9)		
Both	1/122 (0.8)	2.0 (1.5-2.3)			1/122(0.8)	1.5(1.3-2.1)		

OR=odd ratio, CI=confidence interval, X^2^ =chi square, B19V=Human parvovirus B19

## Discussion

The findings of this present study revealed 44.3% B19V IgG prevalence and 41.8% B19V IgM prevalence among the study population. The prevalence as indicated by B19V IgG is similar to the 41.5% reported in Northern Nigeria (Jegede et al., 2014), 52.5% reported in North Central Nigeria (Okojokwu et al., 2018) and 55% reported in Tanzania (Mirambo et al., 2017) but higher than 20% (Abiodun et al., 2013) reported in Southwest Nigeria. The increased prevalence from Southwest Nigeria could be related to time lag since the research was conducted about 10 years ago. This suggests that the prevalence of B19V has doubled over the last ten years. The incidence of the disease as indicated by B19V IgM was very high similar to 39.9% reported in India (Kishore et al., 2010) but higher than 13% reported in Central Nigeria (Emiasegen et al., 2011) and 9.5% in Southwest Nigeria. This disparity may not be unconnected to seasonal peaks variation and the evolving epidemiology of the virus. It has been reported that the peak period of B19V is usually late winter and spring with 75% of IgM-positive cases recorded between January and June (Enders et al., 2007; Crowcroft *et al.*, 1999; Bremner *et al.*, 1994). This present study was in like manner conducted between January and May, 2019. It is also possible that the year 2019 when the study was conducted coincides with the epidemic year of *B19V*. This argument is backed up by the report of Ender and colleagues (2007) which suggested that the epidemic cycle of *B19V* appears to be around 4 years with 1-2 epidemic years followed by 2 or 3 years of less frequent rates of infection. w. However, the *B19V* IgG/IgM co-prevalence which Mirambo *et al.*, referred to as true indicator of incidence was 28.7% in this present study. The prevalence reported is significant because 44.3% of the population do not have antibodies against *B19V* as such if they get infected during a first or second trimester of pregnancy, they may develop complications.

Similar to the findings in North central Nigeria (Emiasegen *et al.*, 2011; Okojokwu *et al.*, 2018), the highest prevalence was recorded among the age group >36 years but contrary to their report, prevalence of IgM was also highest in the lower age group in this study. The reason for this may not be unconnected to cultural and behavioral diversity in different locations of the country. Similar to the report by Abiodun *et al*. (2013) there was no association between age, Incidence (IgM) and prevalence of *B19V* (IgG) recorded in this study.

Seventy-one percent (36/51) of subjects with recent infections as indicated by the presence of IgM where still within the first or second trimester; a high-risk stage for *B19V* complication in pregnant. Prevalence of *B19V* increased progressively with number of parity while prevalence of *B19V* IgM decreased with increase in parity. Contrary to the findings of Okojokwu *et al*. (2018), we found no association between parity and prevalence of *B19V*. There was an association between history of blood transfusion and the prevalence of both IgG and IgM (P = 0.04 and 0.05 respectively). Based on the association recorded, IgG had an odds ratio of 2.1 (95%CI 1.7-2.3) which indicates that those who had *B19V* had a 2.1 times likelihood of haven been exposed to blood transfusion. In like manner, IgM had an odds ratio of 2.5 (95%CI 1.8-2.9) also implying 2.5% likelihood of haven been exposed to blood transfusion. This finding corresponds with previous reports (Heegaard and Brown, 2002; Ender *et al.*, 2007) that documented an association between Blood transfusion and prevalence.

It is hereby recommended that *B19V* screening be included as a criterion for blood donation and transfusion. An association was recorded between history of pregnancy related complications and prevalence of both IgG and IgM (*P*= 0.04 and 0.05 respectively). These complications include Miscarriages and still births. Their odds ratio suggests that women who had suffered miscarriage and still birth had greater than two times likelihood of have been exposed to *B19V* infection. This finding deferred from the report of Okojokwu *et al*. (2018), who reported no association between miscarriage, still birth and prevalence of *B19V*. This is probably due to sociocultural variations between the two study locations. There was no association recorded between parity, number of pregnancies, occupation, marital status and prevalence of *B19V* IgG and IgM. The high incidence rate recorded within a population with a high percentage of susceptible individuals is indicative of an epidemiologic risky situation requiring a strategic intervention plan.

## Conclusion

In the present study, we recorded a 44.3% B19V IgG and 41.8% B19V IgM prevalence rate leaving 44.3% of the population with the potential of getting infected with the virus. The findings point to a possibility of association between history of blood transfusion, history of complication and both prevalence and incidence of B19V. This is indicative of an urgent need for a B19V intervention plan which should include routine laboratory testing and immunization against B19V among pregnant women or intending mothers.

**List of Abbreviations:**
B19V: - Human Parvovirus B19
IQR: - Interquartile range
OR: - Odd Ratio
NIHF: - Non-immune hydrops fetalis
CI: - Confidence interval
